# The Role of Resilience for Migrants and Refugees’ Mental Health in Times of COVID-19

**DOI:** 10.3390/healthcare9091131

**Published:** 2021-08-30

**Authors:** Sara Solà-Sales, Natalia Pérez-González, Julie Van Hoey, Isabel Iborra-Marmolejo, María José Beneyto-Arrojo, Carmen Moret-Tatay

**Affiliations:** 1 Mind, Emotion and Behavioural Research Laboratory (MEB Lab), Faculty of Psychology, Universidad Católica de Valencia San Vicente Mártir, 46100 Valencia, Spain; sara.sola@mail.ucv.es (S.S.-S.); natalia.perez1@mail.ucv.es (N.P.-G.); julie.vanhoey@ucv.es (J.V.H.); isabel.iborra@ucv.es (I.I.-M.); mariajose.beneyto@ucv.es (M.J.B.-A.); 2 Dipartamento di Neuroscienze, Salute Mentale e Organi di Senso (NESMOS), Faculty of Medicine and Psycology, Università Sapienza di Rome, 00187 Rome, Italy

**Keywords:** resilience, migration, refugees, mental health, fear, COVID-19

## Abstract

Migrants and refugees need international protection, particularly during a crisis such as the current health pandemic. The aim of this research was to examine the mental health and attitudes towards COVID-19 in migrants and refugees compared to the general Spanish population. Moreover, the nature of resilience was examined as a mixed component though life experiences. For this proposal, an interview was carried out in a sample of 245 participants who volunteered to participate in the study. The sample was divided into Spanish non-migrants, Spanish migrants, non-Spanish migrants and refugees. Attitudes towards COVID-19, resilience (based on BRCS) and mental health (based on DASS-21) were measured. The results obtained can be described as follows: (i) Migrant participants indicated worse mental health than non-migrants, and within the migrant group, refugees presented worse scores; (ii) No differences were found in attitudes towards COVID-19 in any of the subgroups; (iii) A moderating effect of group was found for the relationship between resilience and mental health but not between resilience and fear of COVID-19. These results might be of great interest in making visible the vulnerability of migrants and specifically refugees, and the proposal of intervention programs based on resilience training.

## 1. Introduction

The health crisis has brought a social and economic collapse. Vulnerable groups may suffer even more complicated and aggravated situations, such as the migrant population and, particularly, refugees. According to the WHO (2020), a large body of barriers and challenges emerged, including loss of livelihoods and stigmatization. At a psychological level, mental health is a sensitive variable for this population. Isolation could emotionally reactivate a traumatic experience in which fundamental rights were truncated, as well as other psychological problems such as depression, anxiety, fear and confusion, among others [1]. Moreover, a fear of disease has been linked to mental health, which has also been reported in refugee cases in the literature [2,3,4,5].

During the first wave of COVID-19, a total of 168 countries closed their borders, resettlement movements were halted and the underlying causes of conflicts remained unresolved (UNCHR, 2020). CEAR (2020) also provides alarming data: countries such as Spain only accepted 5.2% of asylum applications compared to the European average of 31%, and one out of every fifty-five individuals die trying to cross the Mediterranean Sea. This situation should be considered a priority; however, other migrant profiles may also be considered as being vulnerable to the effects of the COVID-19 pandemic.

At the time of the current research, Spain was between the second and third wave of COVID-19. One year after the pandemic, the economic effects were far from recovering. While social restrictions have been relaxed after the lockdown, the economic effects are still far from being diluted, with a possible relapse of the economy. This situation of uncertainty is clearly one of the greatest issues for the country. In particular, it should be noted that the migrant group constitutes an important part of the country’s labour force. According to the Encuesta de Población Activa (2020), the increase in unemployment during the first quarter of 2020 barely reached 0.4% among the national population. However, this percentage reached 17.7% for migrants (up to 22.5% for those individuals with dual nationality).

Beyond the economic consequences, migrants have suffered from other additional effects associated with the measures to curb the virus that have been adopted so far. In this way, the literature points out [6] how necessary it is to assess the real impact, as most of these effects remain unconclusive. Furthermore, this valuable information can help the authorities to adopt evidence-based measures. While many individuals struggle with issues related to fundamental rights, mental health issues seem to have been pushed into the background. In this scenario, a variable of interest is the perception of information regarding the COVID-19 virus. Some authors have found that migrants with high levels of resilience reported lower perceived stress [5]. Although considering oneself informed about COVID-19 is related to a fear of illness, other personal variables might interfere [7]. In this way, the concept of resilience might be of interest.

Several studies have agreed that resilience exerts a protective effect to stressful events [8], concluding that high levels of resilience are related to a lower tendency to develop psychological disorders [9]. Resilient individuals have been described through four pillars: social competence, problem solving, autonomy and positive expectations for the future [10,11]. However, the term resilience is difficult to conceptualize, being addressed from different perspectives in the literature, such as a personality trait or the influence of personality traits and coping experiences [12,13,14]. Understanding resilience as an inherent characteristic of the personality leads to an overlap with other concepts such as resistant personality or hardiness. This approach has reached to such an extent that, for most, both concepts have been considered as interchangeable terms with resilience. For others, resilience could be a mixed component with the environment. Despite this conceptualization limitation, the literature is robust on the protective effect of resilience on mental health [7,15,16].

**Figure 1:**
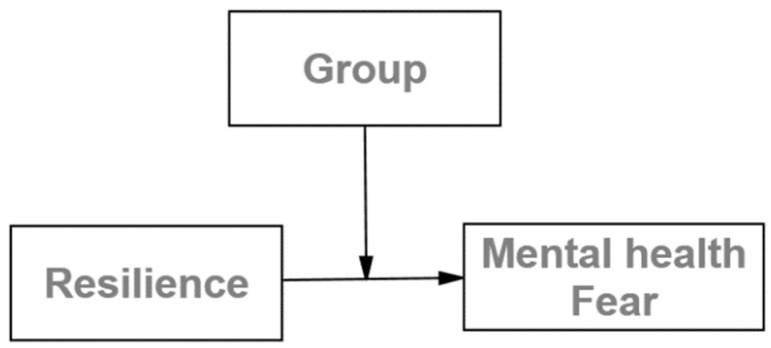
Theoretical model proposed on the moderation effect of migration situation for the relationship between resilience and mental health, as well as fear of COVID-19.

Migrants and refugees with higher resilience scores would be expected to have lower levels of psychopathological distress and, in other words, better mental health [5,15]. However, other literature does not support this relationship [16]. As mixed results have been found in this field, research on this front is of interest. If the relationship between the fear of COVID-19 or mental health with resilience is moderated by group (migrant versus non-migrant individuals), this might shed light on the mixed component of resilience, as proposed in Figure 1. Consequently, these results might be of interest for prevention and intervention programs, as a dynamic variable can be trained, though a trait cannot.

This research aims to examine attitudes towards COVID-19, as well as mental health variables related to migrant individuals and refugees in Spain. A second proposal is to study the nature of resilience in this context. A moderation model is proposed across the relationship between mental health and fear of COVID-19 with resilience across groups of migration or non-migration individuals.

## 2. Method

### 2.1. Materials

After a battery of questions on sociodemographic data, three questionnaires were employed to address the proposals of this study. It should be noted that these questionnaires were not adapted for many of the participant backgrounds under study. However, given their fluency in Spanish and the good psychometric properties of the scores collected, they were considered in the current study.

First, questions developed to measured attitudes towards the COVID-19 outbreak on a Likert scale from 1 to 10 (as reported in previous literature [7,17]) were selected, and the first questionnaire was readapted. These questions are sorted as follows:

(i) I fear COVID-19
(ii) I consider that I have correctly informed myself about COVID-19
(iii) I am concerned about the economic impact that this pandemic may have in my country.

Secondly, the DASS-21 to measure mental health was selected [18]. The Spanish adaptation of the Depression, Anxiety and Stress Scale was employed [19]. A total of 21 items under a Likert type from 0 to 3 were answered by participants. Previous studies have validated it in other samples and found its internal consistency to be between 0.70 and 0.89 [20,21]. Lastly, a Spanish adaptation of the Brief Resilient Coping Scale (BRCS) [22] was employed from the original version [23].

Internal consistency was examined across groups. Adequate values were found for the BRCS scale (α = 0.675 in the migrant group and α = 0.719 in non-migrants). In relation to the DASS-21, once again, the subscales showed adequate values for internal consistency, both for depression (α = 0.818 in the migrant group and α = 0.802 in non-migrants), anxiety (α = 0.811 in the migrant group and α = 0.735 in non-migrants) and stress (α = 0.855 in the migrant group and α = 0.855 in non-migrants).

### 2.2. Procedure

Participants were recruited through a snowball sampling procedure. A total of four researchers conducted the interviews. Once face-to-face or video call contact was established, the information related to data protection and digital acceptance was read, as informed consent. After this step, each of the parts was explained and the questions were read, resolving any doubts or questions. The interviews lasted between 20 and 30 min per participant and took place between January and May 2021.

### 2.3. Participants and Ethics

A sample of 245 participants volunteered to participate in the study, with an age mean 34.95 years (SD = 11.75). The sample was subdivided into two sub-samples: migrant (41.6%) and non-migrant (58.4%) participants. For the first sub-sample, 45.1% were men and 53.9% women, while in the second, 34.3% were women and 65.7% men. For the non-migrant sample, 7% had reached basic studies, 40.6% secondary level and 52.4% a higher education level, while in the migrant sample 18.6% reported basic studies, 64.7% secondary studies and 16.7 higher education. This subsample was subdivided again into: non-Spanish migrants (44.1%), Spanish migrants (27.5%) and refugees (28.4%). All participants had migrated between 1991 and 2021. The study was carried out in accordance with the Helsinki Declaration. Thus, to participate in the different studies, all participants gave informed consent (approval of the committee UCV/2020-2021/065). The study was carried out in Spain and in the Spanish language. The inclusion criteria were: (i) be over 18 years old; (ii), have a high understanding of Spanish.

### 2.4. Data Analysis

All the statistical analyses were performed using the software IBM SPSS 21 and JASP. Assumptions were checked to ensure the adequacy of the analyses: normality, linearity and correlation between variables. The first strategy was to study differences across groups, which was related to the first proposal of the study. In this way, comparisons between groups under study were carried out through parametric and non-parametric approaches. After partial groups correlations among variables of interest, the second strategy was to examine the second proposal of the study though a moderation analysis under a Process macro for SPSS (Hayes, 2015). In this way, regression-based mediation procedures were executed employing bootstrapping procedures.

## 3. Results

**Figure 2:**
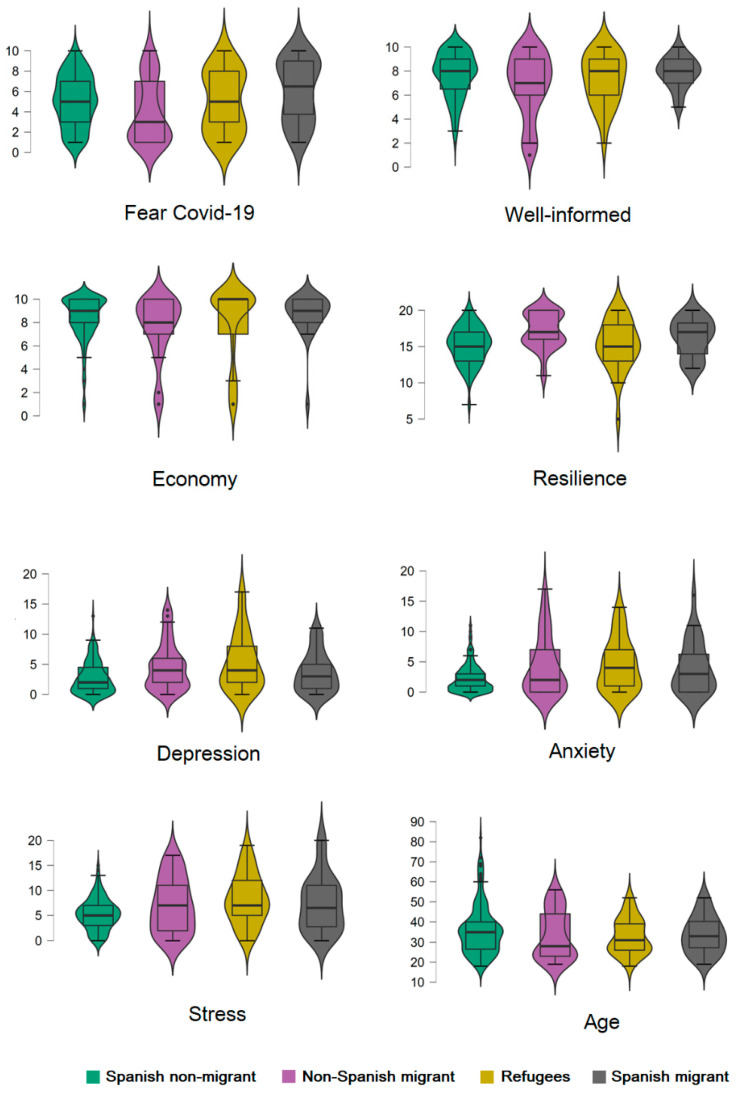
Boxplots on the variables under study across groups.

First, participants were examined across two categories: migrant and not migrant groups. Concerning the comparison of the first two groups, Table 1 shows the Student’s *t*-test for independent samples, with effect sizes and confidence intervals. The migrant group was divided into three other groups: refugees, non-Spanish migrants and Spanish migrants (see Figure 2).

A non-parametric approach was performed for the migration subgroups using the Kruskal–Wallis test. This showed that the differences depicted in the box and whisker plots were statistically significant (*p* < 0.05) except for the variables Well-Informed, Economy and Anxiety (all *p* > 0.05). Partial order correlations were carried out across the main variables of interest, controlled by migration group (Migrant versus non-Migrant), as depicted in Table 2.

**Figure 3:**
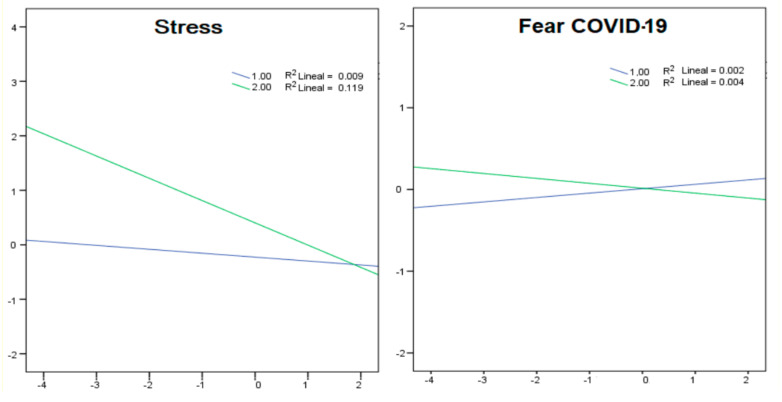

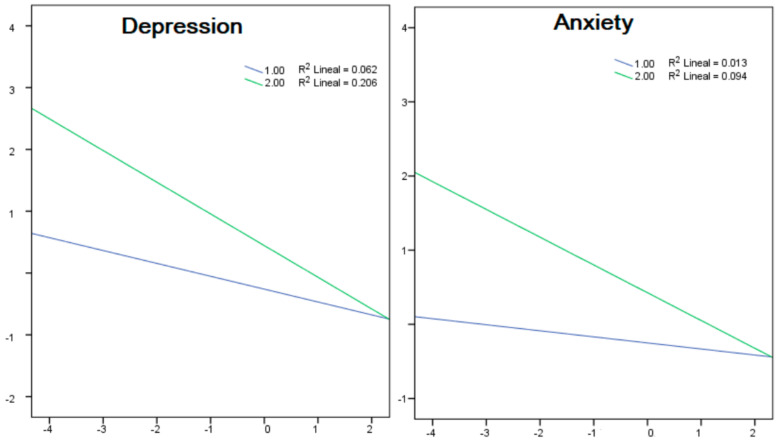
Calculation of the moderation model for resilience across DASS-21 and Fear of COVID-19.

A moderation model was carried out where it was stipulated that the role of resilience on mental health was moderated by circumstances, in this case, having not migrated. The predictive models were statistically significant for all DASS-21 subscales. However, this model was not statistically significant for the variable Fear of COVID-19 (*p* > 0.05). The increase of R^2^ was 0.02 for Anxiety (*p* < 0.05), Depression (*p* < 0.05) and Stress (*p* < 0.001). Table 3 depicts the confidence interval (CI) that at 95% was statistically significant with a confidence interval excluding the zero value, by reporting both lower (LLCI) and upper levels (ULCI) for all of the four analyses. Figure 3 shows the stipulated moderation.

## 4. Discussion

Human beings historically have moved to cover their basic needs, often related to survival, as in the case of refugees. As a part of this process, several changes occur in terms of social or psychological variables, among others. This process is even more complicated in a health crisis such as the current pandemic [24] where stress, anxiety and depression can be increased [15].

This study had a twofold objective. Firstly, it sought to examine differences between the general population and different types of migration. The reference group was the Spanish non-migrant group, and their responses were compared with other Spanish adult migrants, non-Spanish migrants and the most vulnerable group, refugees. The second goal was to examine the nature of the resilience variable.

First, the results showed the worst scores for migrant participants, and within this group, refugees had the worst scores. This highlights their vulnerability as described in previous literature [25,26]. Attitudes towards COVID-19 did not show statistically significant differences, nor did they correlate with resilience in the current study. Two possible explanations are proposed. First, that the migrant group has a larger repertoire of coping strategies to deal with a stressful situation such as the COVID-19 restrictions. This would be supported by a higher score on resilience. The second explanation is bound by one of the main limitations of the study. The attitude variables were measured with only one item, which could introduce some bias, as described in the previous literature employing these measures [7,15]. Furthermore, it must be considered that the reality for these participants is very complex and different cultural variables may intervene in this process. Therefore, more research on the subject is necessary. This situation was clearer for the relationship with other constructs in the study. In this case, resilience did inversely correlate with DASS-21, as expected by the previous literature on the robustness of this relationship [27]. Likewise, it is not surprising that resilience has been considered a protective factor in many fields, such as mental health [28].

On the other hand, by finding such a moderation pattern between resilience and mental health, a mixed component of resilience would be supported [14,29]. This is not only of interest for its theoretical implications but also for its practical implications [30,31]. If resilience can be modified though life experiences, this gives rise to action by public health professionals. It should be noted that resilience has been shown in the literature to play a protective role in mental health [23], therefore, these results would support the possibility of resilience training, especially for vulnerable groups. Moreover, this result is consistent with other recent findings. More precisely, a study comparing migrant versus non-migrant populations after the COVID-19 outbreak showed that migrants’ resilience scores were more resilient than non-migrants in the presence of trauma [16]. According to the authors, this suggests that migrants show a resilience response to adversity, especially in the presence of trauma, but by using cross-sectional data, it is difficult to delineate whether this effect is because these groups of migrants can “walk away” from trauma or because they can adapt themselves better to adversity.

This study has several limitations, highlighting two methodological issues of interest. First, the questionnaires were not adapted to the populations of origin, only to the Spanish population. However, all participants had a high level of Spanish and internal consistency was not affected, all groups were like the Spanish group, so no such effect is expected. On the other hand, it should be noted that the sample size should be increased to improve statistical power. This limits the generalizability of the results. Nevertheless, given the characteristics of the sample, other works in the literature with samples of specific characteristics were taken as a reference [32,33]. Future research should review this possible methodological bias. Lastly, the cross-sectional design of this study has several drawbacks as described above. This type of work would benefit from longitudinal replications to establish systematic replications on the possible origins of differences in resilience between groups.

## 5. Conclusions

The aim of this research was to examine the mental health and attitudes towards COVID-19 of migrants and refugees compared to the general Spanish population. Moreover, the nature of resilience was examined as a mixed component that can be influenced by life experiences. The results obtained can be described as follows: (i) Migrant participants presented worse mental health than non-migrants, and within the migrant group, refugees presented worse scores; (ii) No differences were found in attitudes towards COVID-19 in any of the subgroups; (iii) A moderating effect of group was found for the relationship between resilience and mental health, but not between resilience and fear of COVID-19.

These results might be of great interest for making visible the vulnerability of migrants and, specifically, refugees, and, at an applied level, to implement intervention programs based on resilience training.

## Figures and Tables

**Table 1:** Independent Samples *t*-test across migrants and non-migrants.

					95% CI for Cohen’s d	
Variable	Migrant	Non-Migrant	*p*	Cohen’s d	Lower	Upper
Fear COVID-19	4.81 (3.16)	5.09 (2.51)	0.434	0.102	−0.153	0.356
Well-informed	7.23 (2.24)	7.66 (1.88)	0.106	0.210	−0.045	0.465
Economy	8.02 (2.67)	8.35 (1.94)	0.279	0.141	−0.114	0.395
Resilience	16.38 (2.88)	15.17 (2.56)	<0.001	−0.448	−0.704	−0.190
Depression	4.55 (4.02)	2.73 (2.65)	<0.001	−0.554	−0.812	−0.295
Anxiety	4.30 (4.47)	2.31 (2.31)	<0.001	−0.589	−0.847	−0.329
Stress	7.48 (5.31)	5.28 (3.09)	<0.001	−0.527	−0.785	−0.269

**Table 2:** Partial Pearson’s Correlations across the whole dataset, controlled by migration group (Migrant versus non-Migrant).

Variable	1	2	3	4	5	6	7
Fear COVID-19 (1)	—						
Well-Informed (2)	0.133 *	—					
Economy (3)	0.170 **	0.124	—				
Resilience (4)	−0.007	0.097	0.096	—			
Depression (5)	0.016	−0.080	0.027	−0.357 ***	—		
Anxiety (6)	0.197 **	−0.002	0.111	−0.224 ***	0.603 ***	—	
Stress (7)	0.146 *	0.011	0.018	−0.234 ***	0.606 ***	0.733 ***	—

* *p* < 0.05, ** *p* < 0.01, *** *p* < 0.001.

**Table 3:** Conditional effect of resilience on mental health at values of the moderator. Effects, standard error (SE), statistical significance and lower and upper (LLCI and ULCI) level.

Model		Coeff	SE	*t*	*p*	LLCI	ULCI
Depression	Constant	0.0324	0.0609	0.5315	0.5955	−0.0876	0.1524
	Group	0.7044	0.1329	5.2992	<0.001	0.4425	0.9662
	Resilience	−0.3349	0.0624	−5.3668	<0.001	−0.4578	−0.212
	Interaction	−0.3044	0.13	−2.341	0.02	−0.5605	−0.0483
Anxiety	Constant	0.0312	0.062	0.5025	0.6158	−0.091	0.1533
	Group	0.6768	0.1392	4.8603	<0.001	0.4025	0.9511
	Resilience	−0.2036	0.059	−3.4526	0.0007	−0.3197	−0.0874
	Interaction	−0.2928	0.127	−2.3056	0.022	−0.5429	−0.0426
Stress	Constant	0.0358	0.0603	0.5938	0.5532	−0.083	0.1547
	Group	0.6289	0.1324	4.7502	<0.001	0.3681	0.8896
	Resilience	−0.2121	0.0557	−3.8074	0.0002	−0.3218	−0.1024
	Interaction	−0.3366	0.1138	−2.9569	0.0034	−0.5609	−0.1124

## Data Availability

Data would be available on request.
